# Roadmap for sexual medicine: Agenda for Indian Psychiatric Society

**DOI:** 10.4103/0019-5545.43619

**Published:** 2008

**Authors:** T. S. Sathyanarayana Rao, Ajit Avasthi

**Affiliations:** Department of Psychiatry, JSS Medical College Hospital, Ramanuja Road, Mysore - 570 004, India; 1 Post Graduate Institute of Medical Education and Research, Chandigarh - 160 012, India

For life to be born and perpetuate, union of the female and male is quintessential across species. For thousands of years, sexual pleasure has been depicted in various hues; darker and lighter, enraging and soothing. Sex has been revered, worshipped, followed and yet tabooed, looked down upon, considered a vice to protect oneself from. No single thing about life is so variedly thought of as is sexual union. Artists have symbolized sex in various forms expressing through words, paintings, and sculptures. Nonetheless, sex continues to be talked about in hushed tones, not to be discussed beyond closed doors.

In such a scenario, the good and ills about carnal union remain undisclosed and many a suffering unknown. Men and women undergo great distress from sexual problems but help -seeking is vehemently avoided. When any help is sought, it is from friends, local healers and self proclaimed doctors who promise guaranteed cure within 24 h. With local media flashing such attractive advertisements, the sufferers easily fall prey. To say the least, most of the people suffering from sexual problems never reach the ‘doctor’ to seek medical advice. This is especially true for our country.

Against this background, the initiative taken by the Indian Psychiatric Society to provide a special platform for sexual medicine is rather commendable. The Continuing Medical Education (CME) session that was held in Ahmedabad in the year 2008 amassed expert opinions and brilliant suggestions. This effort has paved the way further and strengthened the foundation for the science of sexual medicine in India. On the clinical front, the Indian Psychiatric Society (IPS) has already taken an extremely crucial step, i.e. the IPS has generated the clinical practice guidelines[1] for sexual disorders. These clinical practice guidelines provide a comprehensive review of Indian research and moreover, include management algorithms for various sexual disorders. This effort is definitely bound to make a significant impact on the clinical practice in this field. Research and management guidelines are still conspicuously absent for paraphilias and gender identity disorders.

Where does sexual medicine stand in terms of general medicine and psychiatry and behavioral sciences? General health is reflected through various biological functions of the human body such as appetite, sleep and libidinal drive. Sexual health forms an integral part of general health with both physical and mental components to it. Both physical and mental fitness ensure healthy sexual life.

Physical illnesses such as diabetes mellitus and hypertension affect various systems including cardiovascular and nervous (including autonomic) systems. With these vital systems playing important role in the execution of sexual functions, it is natural that sexual health is affected adversely. In other words, sexual health is closely intertwined with physical health. So, the integration of sexual medicine with other branches of medicine is absolutely essential, but has not yet occurred in a significant way.

The importance of sexual health in psychiatry, to say the least, is immense. Primary sexual problems affect overall mental health of the individual and the reverse holds as much truth. Anxiety disorders and anxious predispositions have enormous bearing on sexual functioning, whereas changes in libido accompany mood disorders. Not just that, these disorders are of relevance but also the psychopharmacological agents cause a myriad of sexual side effects.

Let us have a status check on where we stand currently in this field and where we lack. Sexual problems are highly prevalent in men and women, but are highly under-recognized and under-reported. Majority of epidemiological studies of erectile dysfunction and premature ejaculation are conducted on clinical populations. The numbers generated are far from reality. In fact, what we see in our clinics is just the ‘tip of the iceberg’. Studies primarily focus on patients attending psychiatric clinics and a few psychosexual clinics, which generate break-up of various psychosexual disorders. For instance, it was found that amongst males with psychosexual dysfunction, 40% had erectile dysfunction, another 40% had combination of erectile dysfunction and premature ejaculation, only 20% had premature ejaculation alone.[2]

Studies restricted to psychiatric and special clinics do not give an estimate of those patients who present primarily with psychiatric disorders. Akin to what happens in such research, in clinical practice too, sexual dysfunctions go unrecognized in patients with mental disorders. To add to the problem is the array of sexual side effects of various psychotropics. These side effects pose a great challenge in treatment of these patients. We need to study the extent of these problems systematically so as to be able to devise solutions. The studies, in due course of time should be community based which to say the least give us a glimpse of the reality.

Over the years, various studies have been carried out on ‘dhat’ syndrome. Bhatia *et al.*[3] found that two-thirds of the patients with potency disorders had ‘dhat’ syndrome. A questionnaire has been developed on sex knowledge and attitude (SKAQ) in Hindi for North Indian population.[4] Similarly, Sharan *et al.*[5] developed a 13 item semi-structured interview schedule for assessment of dhat syndrome. Development of such culturally oriented instruments is crucial to the research of sexual behaviors and disorders.

To rightfully emphasize again, to begin with, research in this area should include development of instruments, assessment of attitudes and beliefs, prevalence rates of various disorders. It should further encompass trials of pharmacological and non-pharmacological modalities to treat these disorders.

Steps in this direction have already been taken. Since 1980s, workers like Bagadia *et al.*[6] have shown the usefulness of non-pharmacological measures in treatment of impotency and pre-mature ejaculation. Also, manual providing algorithms for erectile dysfunction, PME, dhat syndrome and homosexuality has been made.[7] But, there is dismal research in relation to the use of pharmacological agents for sexual dysfunction from India. A handful of studies have been done and these are mainly open trials conducted with small sample sizes.

Needless to say, studies comparing non-pharmacological and pharmacological treatment are few and far in between. Prusty and Rath[8] found that patients treated with behavior therapy showed lasting treatment effect but poor patient tolerability as compared to those treated with clomipramine. Instead of drawing immediate conclusions, such seminal work should be used as the stepping stone to generate rich evidence base. Only then, can we formulate truly evidence based treatment plans.

So, with the knowledge of the already existing data base, as mentioned earlier, it is strongly recommended that reliable estimates be generated of primary sexual disorders and those co-morbid with psychiatric disorders. This should be done across various settings, viz., specialty clinics, general psychiatry clinics, general medical clinics and the community.

Also, special efforts need to be made to study cultural aspects of sexual health and sexual disorders. Genesis and administration of culturally relevant instruments is one of the crucial steps. Understanding the cultural underpinnings of various disorders and formulating culturally relevant treatment plans must be the goal.

To further the science of sexual medicine, it is most crucial that the trainee psychiatrists are amply exposed to clinical experience in the field. Supervised training in thorough case work up and management of patients attending psychosexual clinics should be mandatory. Manuals as the one mentioned before[7] would come as an aid to the trainee doctors. Basic training in sexual medicine at the undergraduate level which is completely lacking needs to be introduced. Regular Continuing Medical Education programmes, seminars and discussions in the fraternity would provide the required momentum. In the long run, as steps to further the science of sexual medicine shall be taken, involving the community through sex education and such other programmes would be inevitable.

As mentioned earlier, sexual health has twin facets – physical and mental. But the individual patient is torn between the various specialties, i.e. urology, neurology and psychiatry. It is time that with the development of sexual medicine, the psychiatrist acts as the coordinator. He should take up the responsibility of integrating the various fields and offer comprehensive services in the field of sexual health and medicine. One way of achieving this goal would be to establish multispeciality sexual clinics, affiliated to teaching institutions with comprehensive liaison activities.
